# Incidence of Greenlandic stroke-survivors in Greenland: A 2-year cross-sectional study

**DOI:** 10.3402/ijch.v72i0.22626

**Published:** 2013-11-22

**Authors:** Karen Bjorn-Mortensen, Folmer Lynggaard, Michael Lynge Pedersen

**Affiliations:** 1 Queen Ingrid's Health Center, Nuuk, Greenland; 2 Greenland Center for Health Research, University of Nuuk, Nuuk, Greenland; 3 Department of Internal Medicine, Queen Ingrid's Hospital, Nuuk, Greenland

**Keywords:** stroke, Greenlanders, incidence

## Abstract

**Objectives:**

To estimate age- and gender-specific incidence rates among Greenlandic stroke-survivors.

**Study design:**

The study was performed as a cross-sectional observational study.

**Methods:**

All Greenlandic patients admitted to Queen Ingrid's Hospital (QIH) with stroke in 2011 and 2012 were included in the study. Data were obtained from patient files and the Central Civil Registration System. Age- and gender-specific incidence rates were estimated as cases/100,000 adults/year. Direct age-standardized incidence rate was calculated using the WHO 2000–2005 population as the standard.

**Results:**

In 2011 and 2012, 156 cases of stroke were registered, 72 (46.2%) males and 84 (53.8%) females. The overall incidence rate of stroke was 155/100,000 person-years (95% CI 121–190), with ischemic stroke accounting for 89.1% of these. No significant differences were seen between men and women. Direct age-standardized incidence rate was 149/year/100,000 (95% CI 192–264). Median age at time of diagnosis was 60 years (interquartile range 53–69).

**Conclusions:**

This study reports an age-standardized all-stroke incidence rate of Greenlandic stroke-survivors in Greenland within the wide range as incidences in Western Europe. A noticeable difference when compared to Denmark was that male and female incidence were approximately the same, and that incidence rates were high in the younger age groups. The majority of strokes were of ischemic origin.

Annually, more than 15 million people suffer a stroke of either ischemic or haemorrhagic origin worldwide. More than 60% of these either die or become dependent on others, making stroke the second leading cause of death (1).

A low frequency of acute myocardial infarction but a high frequency of stroke has been reported in Greenland (2,3) as well as among Alaska Inuit and Alaska natives (4,5). Similarly, a low mortality rate from ischemic heart disease and a high mortality from cerebrovascular disease have been reported among Greenlanders (6) and in the Canadian Arctic population (7–9). Earlier reported stroke frequencies were limited to the population of Upernavik (2) and Nuuk (3) or based on death certificates (6). Computer tomography (CT) scans have only been available in Greenland since 1997 and not previously been performed on a regularly basis. The current prevalence of stroke in Greenland is unknown.

From 1 January 2010, a new strategy concerning stroke patients in Greenland was implemented. The strategy aims that all patients suspected of a cerebral stroke are transported to Nuuk for a cerebral CT scan, ultrasound examinations of the carotic arteries, event recordings, echocardiographies, blood samples and initiation of rehabilitation. Since 2010, data have been available on all-stroke patients surviving stroke long enough to be transported to Nuuk in Greenland. Since the implementation of a new strategy will incur some delay, data can be considered representative after 2011.

The aim of this study was to estimate the age- and gender-specific incidence of stroke-survivors in Greenland.

## Materials and methods

### Study design and period

The study was carried out as a cross-sectional observational study, including all patients admitted to Queen Ingrid's Hospital (QIH) in Nuuk with stroke in 2011 and 2012.

### Setting

Approximately 56,000 people live in Greenland's 18 towns and 60 settlements. Of these, 88.7% was born in Greenland (10).

For most Greenlanders, contact with the healthcare system is through a local nurse or a doctor at the nearest medical clinic, while in Nuuk a health clinic provides primary care and serves as an entrance to QIH, the central hospital for all of Greenland. Only QIH in Nuuk has a CT scanner and the medical expertise to perform ultrasounds, echocardiographies and event recordings on a regular basis.

### Study population

Only Greenlanders, defined as persons born in Greenland, were included in the study. All patients discharged from QIH with the International Classification Diagnoses of subarachnoid haemorrhage (SAH) (I60), intracerebral haemorrhage (I61), cerebral infarction (I63) and cerebral apoplexy (I64) were identified from the Greenlandic Patient Discharge Registry.

If for obvious reasons the diagnoses were wrong, for example, cerebral tumours or abscesses, or if the onset of symptoms occurred before 2011, these patients were excluded. Patient files for all patients discharged in the spring of 2013 were reviewed and if the onset of symptoms was in 2012, these patients were included.

### Data sources

Data sources were linked using the unique 10-digit Civil Registration Number given to all citizens of Greenland at birth. Data on place of birth were obtained from the Central Civil Registration System. Details of the onset of symptoms, date of admittance and examination results for each patient were obtained from patient files. In addition, the Chief Medical Officer in Greenland was contacted in order to obtain data on deaths from stroke in the study period from the mortality statistics.

### Definitions of variables

Stroke was defined according to the WHO (11) as a sudden focal or global neurological impairment lasting >24 hours or leading to death. The definition includes ischemic infarction, intracerebral haemorrhage and SAH. Transient ischemic attacks lasting <24 hours are excluded in the definition. Both first-ever and recurrent strokes were included in the study. A stroke was considered recurrent if medical records showed evidence of an earlier stroke.

A CT scan was considered normal if either no pathology was found or if no change in earlier known pathology was found.

If the CT scan was normal, the cause was classified according to the discharge diagnosis. If the CT scan was normal, with the patient discharged with a diagnosis of cerebral apoplexy, the cause of the stroke was classified as ischemic.

### Statistical analyses

Age- and gender-specific incidence rates were calculated using the Greenlandic population as per 1 January 2012 (10) as background population. Only persons born in Greenland were included in the population. Chi-square tests were used to compare frequencies. Age was described using medians and interquartile range (IQR). Age medians among groups were compared using Kruskal–Wallis H test. P-values at 0.05 were used as a level of significance. Estimates were calculated with 95% confidence intervals. In addition, direct age-standardized incidence rates were calculated using the WHO 2000–2005 population as the standard (12).

Statistical analyses were performed using SPSS statistical software, version 21 (Norusis; SPSS Inc., Chicago, IL).

The ethics committee for medical research in Greenland approved the study.

## Results

### Study population

First, 193 patients with a relevant discharge diagnosis from QIH in 2011 or 2012 were identified. Of these, 172 had a correct diagnosis, while 21 were excluded due to date of event prior to 2011, wrong diagnosis or because the same patient had 2 identical admission- and discharge dates listed. Of the 172 patients discharged with stroke in 2011 or 2012 from QIH, 156 were Greenlanders. No significant difference were found between the number of patients discharged in 2011 (n=84) and 2012 (n=72), p=0.337. Mortality statistics from the Chief Medical Officer were available for 2011. In 2011, stroke was listed as a cause of death in 17 persons. Four of these were already included in this study.

### Characteristics and examination results

Results are summarized in Table I for both ischemic infarction, intracerebral haemorrhage, SAH and in total. Of the 156 patients, 74 (47.4%) were male and 82 (52.6%) were female. Median age at time of diagnosis was 60 years (IQR 53–69). Patients with an ischemic stroke were older than patients with intracerebral haemorrhage and SAH (p=0.014).

**Table I:** Characteristics of Greenlandic stroke-survivors in Greenland 2011–12

Number of strokes (n)				
Total	172			
Not Greenlandic	16			
Greenlandic	156			

	Ischemic infarction n (%)	Intracerebral haemorrhage n (%)	Subarachnoid haemorrhage n (%)	Total n (%)
Study population	139 (89.1)*	7 (4.5)*	10 (6.4)*	156 (100.0)
2011	74 (53.2)	4 (57.1)	6 (60.0)	84 (53.8)
2012	65 (46.8)	3 (42.9)	4 (40.0)	72 (46.2)
Gender				
Male	64 (46.0)	4 (57.1)	6 (60.0)	74 (47.4)
Female	75 (54.0)	3 (42.9)	4 (40.0)	82 (52.6)
Discharge diagnoses				
DI64: cerebral apoplexy	126 (90.6)	4 (57.1)	1 (10.0)	131 (84.0)
DI63: cerebral infarction	11 (7.9)	1 (14.3)	0 (0.0)	12 (7.7)
DI61: cerebral haemorrhage	2 (1.4)	0 (0.0)	1 (10.0)	3 (1.9)
DI60: subarachnoid haemorrhage	0 (0.0)	2 (28.6)	8 (80.0)	10 (6.4)
Previous stroke				
Total	22 (15.8)	0 (80.0)	1 (10.0)	23 (14.7)
CT scan				
Total	138 (99.3)	7 (100.0)	10 (100.0)	155 (99.4)
Ischemic infarction	107 (77.0)	0 (0.0)	0 (0.0)	107 (68.6)
Haemorrhagic infarction	0 (0.0)	7 (100.0)	0 (0.0)	7 (4.5)
Subarachnoid bleeding	0 (0.0)	0 (0.0)	10 (0.0)	10 (6.4)
Normal	31 (22.3)	0 (0.0)	0 (0.0)	31 (19.9)
Mortality				
Dead <28 days of stroke	4 (2.9)	0 (0.0)	3 (30.0)	7 (4.5)
Median age				
Years (IQR)	62 (54–71)	56 (45–62)	56 (47–58)	60 (53–69)
Incidence rate				
n/100,000 person-years (95% CI)	138 (115–161)	7 (2–12)	10 (4–16)	155 (121–190)

* % of total study population.

CI, confidence interval.

The majority of strokes were ischemic, since 139 patients (89.1%) had ischemic infarction, 10 (6.4%) had SAH and 7 (4.5%) had intracerebral haemorrhage. Recurrent stroke was seen in 23 (14.7%) stroke patients, the majority being from the group with ischemic stroke. No patients had 2 strokes within the study period. CT scans were performed on almost all patients (155 (99.4%), showing ischemic infarction in 107 (68.6%), SAH in 10 (6.4%) and haemorrhagic infarction in 7 (4.5%) of patients. Normal CT scans were found among 31 (19.9%) patients, all from the ischemic group.

### Incidence rates

Age- and gender-specific incidence rates are shown in Table II. The overall incidence rate of stroke among Greenlandic stroke-survivors in 2011–2012 was 155/100,000 person-years (95% CI 121–190) or 203/100,000 person-years >15 years (95% CI 158–248). The highest incidence rate observed was 819/100,000 person-years in the 65–74 years age group (95% CI 525–1,073). No statistically significantly differences were observed between genders (see Table II). Additionally, 13 persons (per year) from mortality statistics could have been included in the calculations if cause of death were correct. This would give an incidence rate of approximately 180/100,000 person-years.

**Table II:** Age- and gender-specific incidence rates of stroke-survivors in Greenland 2011–12

	Male		Female			Total	
							
Age	Incidence/100,000 person-years (95% CI)	n/N	Incidence/100,000 person-years (95% CI)	n/N	p	Incidence/100,000 person-years (95% CI)	n/N
25–34	15 (0–44)	1/3,355	0	0/3,242	0.326	8 (0–56)	1/6,597)
35–44	93 (19–168)	6/3,214	116 (30–202)	7/3,019	0.696	104 (24–199)	13/6,233
45–54	109 (42–177)	10/4,572	228 (126–330)	19/4,172	0.055	166 (81–205)	29/8,744
55–64	634 (416–853)	32/2,522	469 (264–674)	20/2,132	0.285	559 (345–944)	52/4,654
65–74	634 (326–943)	16/1,261	1147 (734–1,560)	29/1,264	0.051	891 (525–1,073)	45/2,525
75–84	1150 (408–1,894)	9/391	614 (162–1,066)	7/570	0.201	832 (258–2,208)	16/961
All>15	188 (145–230)	74/19,707	159 (125–193)	82/25,801	0.347	203 (158–248)	156/38,498
All	143 (152–242)	74/18,791	167 (131–203)	82/24,539	0.339	155 (121–190)	156/50,340

Direct age-standardized incidence rate of all strokes was 149 strokes/100,000 person-years (95% CI 192–264). Type-specific incidence rates of ischemic and haemorrhagic strokes are shown in Table I.

Seven patients (4.5%) in the study group died within the first month of their stroke. Of these 3 (43%) suffered from SAH.

## Discussion

The overall incidence rate of stroke among Greenlandic stroke-survivors in Greenland was 155/100,000 person-years within 2011–2012, with ischemic stroke accounting for almost 90% of these. The highest incidence rates were found among the oldest age groups.

The major strength of this study was that it included all admitted stroke patients to DIH in a specific period of time. Since the new strategy in 2010, ensuring that all patients suspected of a stroke in Greenland were transported to QIH, Nuuk, ideally this would mean that the incidence was identical to the incidence of stroke among Greenlanders in Greenland.

However, the study was based on discharge diagnoses from QIH and therefore has a limitation due to underestimation. First, there is a risk that minor strokes not recognized would not be admitted to QIH. Second, patients with very severe symptoms might die before reaching Nuuk. Third, since identification of patients was based on discharge diagnoses, some patients will be missed, for example, patients who are misclassified or given wrong discharge diagnosis.

Thus, the incidence of stroke is higher (180 versus 155/100,000 person-years) when including data from death certificates. Calculated incidence in this study should therefore be considered a minimum incidence of stroke among Greenlanders in Greenland.

Due to an expected delay in the implementation of the new stroke strategy in 2010, data from 2010 were not used in this study. Also, the small study population limited the accuracy of estimates.

A high frequency of strokes has previously been reported in Greenland (2,3,6). The most recent study on stroke patients in Greenland was based on discharge diagnoses and is the only other study after the introduction of CT scans. The study reported an incidence rate of 35 stroke patients/3 years/13,445 residents of Nuuk (3), which can be calculated as an incidence rate of 86.8 strokes/100,000 person-years. The incidence rate of 155/100,000 person-years found in this study was markedly higher. SAH patients were not included in the 2003 study and incidence would have been higher if these patients were included. Nevertheless, it does not account for the total difference in incidence rates. The best explanation is that the new stroke strategy from 2010, which ensures that all patients, no matter the degree of symptoms, are admitted to QIH, leads to a higher incidence rate.

Stroke incidence rate, whether calculated per inhabitant above 15 years of age or per total population or including inhabitants of all ages (see Table II) compared to that of other countries, was remarkably comparable even if it included mortality statistics (13). Since 14.7% of the patients in this study had had an earlier stroke or transient ischemic attacks, incidence rates of first-ever stroke in Greenland will be even lower. This could partly be explained by the low mean age and life expectancy in the Greenlandic population (10,14). Age-standardized incidence rate was 149/100,000 person-years, which is higher than age-standardized incidence rates reported in a large review (13) from, for example, Denmark (80–106/100,000), France (58–92/100,000), Germany (85/100,000) and Italy (82–113/100,000) but lower or in the same range as incidence rates reported from, for example, Norway (154/100,000), Finland (141–367/100,000), Sweden (83–312/100,000), Portugal (118–261/100,000) and Japan (260–375/100,000). However, the review showed large variations both on a national and international basis.

Stroke incidence rates are generally difficult to compare due to great methodological differences in study designs and populations (15). WHO estimates of stroke are based on mortality reports and the assumption that the proportion that dies of stroke is constant in all countries and thereby extrapolation of survivors can be found. Yet, WHO estimates has been reported and provide reliable and comparable data on stroke after a large review study in 2006 (15).

When comparing the age-specific incidence rates found in this study with WHO estimates of stroke in Denmark 15 or stroke incidence rates reported from Denmark (16,17), a noticeable difference was seen in the younger age groups for both men and women. Stroke incidence rate was markedly higher in Greenland in these age groups. For women, this was true for almost all age groups (15), for example, 116 versus 30 (35–44 years), 228 versus 80 (45–54 years), 469 versus 184 (55–64 years) and 1,147 versus 580 (65–74 years) strokes/100,000 person-years (15). However, in the oldest age group, the incidence rate of stroke among females was lower in Greenland than in Denmark. Life expectancy in Greenland is low, for example, almost 10 years lower compared to Denmark (10,14) and therefore less persons live to experience a stroke at old age.

The incidence rate of SAH in this study was 10/year/100,000, which is approximately the same as reported in an earlier study (18), which was then reported to be 3 times as high as the Danish population. The higher incidence rate of stroke in the younger age groups can, to a minor extent, be explained by the higher incidence of SAH among Greenlanders due to familial aggregation (19), but does not explain why more women experienced a stroke in Greenland compared to Denmark. These findings are supported by earlier reports of higher female incidence of stroke among Alaskan Inuits than among other populations (20,21).

In conclusion, this study reports an age-standardized all-stroke incidence rate among Greenlanders in Greenland within the wide range of incidence rates reported from Western Europe.

The most noticeable difference in stroke among Greenlanders was that both men and women experienced the same number of strokes, making female incidence markedly higher than among other populations. In addition, stroke incidence rates were markedly higher in younger age groups. Stroke patients in Greenland were young at the time of event and the majority of strokes were of ischemic origin. To evaluate trends in Greenlandic stroke incidence and mortality, larger studies are needed in the future.
